# Toward Safer Biotherapeutics: Expression and Characterization of a Humanized Chimeric L-Asparaginase in *E. coli*

**DOI:** 10.3390/ijms26146919

**Published:** 2025-07-18

**Authors:** Alejandro Pedroso, Javiera Miranda, Nicolás Lefin, Brian Effer, Enrique Pedroso Reyanldo, Yolanda Calle, Gisele Monteiro, Adalberto Pessoa, Jorge G. Farias

**Affiliations:** 1 Department of Chemical Engineering, Faculty of Engineering and Science, Universidad de La Frontera, Temuco 4811230, Chile; a.pedroso01@ufromail.cl (A.P.); j.miranda05@ufromail.cl (J.M.); n.lefin01@ufromail.cl (N.L.); 2 Departamento de Ciencias Básicas, Facultad de Ciencias, Universidad Santo Tomas, Temuco 4780000, Chile; 3 Center of Excellence in Translational Medicine (CEMT) and Scientific and Technological Bioresource Nucleus (BIOREN), Universidad de La Frontera, Temuco 4811230, Chile; breeiky@hotmail.com; 4 Comité Científico Araucania Sur, Colegio Médico de Chile, Temuco 4780000, Chile; enrikepedroso085@gmail.com; 5 School of Life and Health Sciences, University of Roehampton, London SW15 4JD, UK; yolanda.calle-patino@roehampton.ac.uk; 6 Department of Biochemical and Pharmaceutical Technology, School of Pharmaceutical Sciences, University of São Paulo, São Paulo 05508-000, Brazil; smgisele@usp.br (G.M.);

**Keywords:** acute lymphoblastic leukemia (ALL), L-asparaginase (ASNase), chimeric, recombinant protein

## Abstract

Acute lymphoblastic leukemia (ALL) is the most common cancer affecting children, making up about 80% of all acute leukemia cases in the pediatric population. While treatment with L-asparaginase (ASNase) has greatly improved survival rates, its bacterial origin often causes immune reactions in some patients, which can reduce how well the therapy works. To overcome this challenge, previous in silico studies designed a humanized chimeric ASNase by swapping out the predicted immunogenic parts of the bacterial enzyme with similar, less immunogenic segments from the human version—while keeping the enzyme’s active site intact. In this study, the chimeric L-asparaginase designed was successfully cloned, expressed, and purified using the *Escherichia coli* Rosetta strain. The production conditions (37 °C, 0.01 mM IPTG, 2–4 h) were optimized, and we purified the enzyme in a single step with nickel-affinity chromatography. The enzyme’s activity was confirmed in vitro, showing that it is possible to produce a functional humanized variant in a bacterial system. These results lay important groundwork for future research to assess the immune response and therapeutic potential of this novel chimeric enzyme.

## 1. Introduction

Acute lymphoblastic leukemia (ALL) is the most common childhood cancer, accounting for 80% of acute leukemias in children. Survival rates have improved dramatically from under 10% in the 1960 s to around 80–90% long-term disease-free survival in developed countries today. However, 10–20% of patients still experience treatment failure due to relapse and chemotherapy resistance. New therapeutic strategies are needed to improve outcomes for this group [1,2].

### 1.1. L-Asparaginase (ASNase): Sources of Obtention, Structural Characteristics, and Mechanism of Action

Leukemic cells, unlike normal cells, present a deficiency in the synthesis of L-asparagine, an amino acid necessary for their development and proliferation. Epigenetic factors such as acetylation and histone methylation affect the synthesis of this amino acid, as well as hypermethylation, in CpG islets, of the promoter of the asparagine synthetase enzyme gene (ASNS), an enzyme that catalyzes the synthesis of L-asparagine. This amino acid is essential for the development and proliferation of leukemic cells and therefore, when this amino acid is depleted, leukemic cells, as an alternative route, acquire L-asparagine from blood plasma [3].

Among the drugs used for the chemotherapeutic treatment of childhood acute lymphoblastic leukemia (ALL) is L-asparaginase (ASNase), an enzyme that has successfully contributed to increase survival rates in this disease [4,5]. Several ASNase formulations are currently available on the market for therapeutic use. There is native ASNase derived from *Escherichia coli* [6], a recombinant preparation of ASNase from *E. coli* [7], and two PEGylated forms of ASNase from native *E. coli* [8,9]. In addition, the ASNase enzyme isolated from *Erwinia chrysanthemi* [10] and a recombinant version of the same [11] are also available. Although these biopharmaceuticals retain the same active ingredient, they differ in aspects such as half-life time, frequency of administration, and the generation of immune response in patients and its intensity.

L-asparaginase is the first therapeutic enzyme with antineoplastic properties that has been extensively studied by researchers and scientists worldwide. It was first observed in 1904. Research on the physiological capabilities of ASNase was ongoing for more than half a century with a real breakthrough in 1922, when Clementi revealed the presence of L-asparaginase in guinea pig blood serum [12]. In 1953, Kidd first described the tumor-inhibitory properties of ASNase, with the observation that lymphoma-bearing mice treated with guinea pig serum experienced rapid and often complete tumor regression [13]. These properties were subsequently attributed to ASNase activity [10,14]. L-asparaginase is prevalent among eukaryotes and microorganisms [15]. The presence of this enzyme has been reported in several organisms, including animals and plants. However, due to the tedious extraction procedures, researchers explored other possible sources such as bacteria, fungi, algae, yeasts, and actinomycetes, as the large-scale production of the enzyme from microorganisms is much easier since the culture conditions can be easily optimized for enzyme production in large quantities. In addition, these microorganisms can be genetically modified in a simple manner, thus inducing an increase in yield, a more stable enzyme, and favorable and more economically viable extraction and purification conditions [16].

These enzymes can be divided into three classes according to their amino acid sequence [17]. The first class, which constitutes the most widely used source, are enzymes from Gram-negative bacteria. L-asparaginase from most Gram-negative bacteria can be classified into two main types: type I and type II L-asparaginase. Type I L-asparaginase is quantitatively expressed and possesses enzymatic activity on the amino acids L glutamine and L-asparagine, whereas type II L-asparaginase possesses high specific activity on L-asparagine and is only induced under anaerobic conditions [17]. Type II L-asparaginase produced from *Escherichia coli* (EcA II) and *Erwinia chrysanthemi* (Er A) has been used as an antitumor agent for the effective clinical treatment of ALL for more than 30 years to date with excellent results [18].

There is a variant of ASNase in plants [19] and another in microorganisms such as the fungus Rhizobium etli [20], which represent the second and third classes, respectively, but neither shows homology with the other or with the other ASNases of bacterial origin. The existence of human L-asparaginase has also been reported, but in spite of presenting the same mechanism of action as the enzyme of bacterial origin, the known human variants are not adequate replacements for the bacterial enzymes used clinically, since they have a very high KM value [21]. On the other hand, as both are orthologous proteins with the same functionality, the human enzyme presents a 19, 84% sequence identity with the *E. coli* variant and 116 immunogenic epitopes less than this bacterial enzyme, which indicates the existence of conserved amino acid sequences in both proteins.

Structurally, in its native conformation, ASNase is characterized by a tetrameric form, although hexameric, dimeric, and monomeric forms have been found in other sources [12,22]. ASNases from *Erwinia* sp. and *E. coli* have similar three-dimensional structures [22]. The *E. coli* ASNase enzyme (ECAII) is chemically known as *E. coli* L-asparagine amidohydrolase and consists of four identical subunits, each consisting of 326 aa and with a molecular weight of 35.6 kDa [22,23]. Characteristic residues of the active center include T12, Y25, S58, T89, D90, and K162, with T12 and T89 being key to the reaction mechanism [22]. It has been reported in the literature that the N- and C-terminal domains of the subunits are involved in the formation of each active site, so ASNase homotetramers are best described as dimers of closely related dimers [22,24]. The purified enzyme exhibits a specific activity of close to 200–400 IU/mg protein [25]. Other properties of *E. coli* ASNase have been determined such as the isoelectric point, which is 4.9 [25], the pH optimum, which ranges from 7 to 8, and the temperature optimum, which is 37 °C [25]. Kinetically, the *E. coli* enzyme is known to exhibit a Michaelis Menten constant (KM) of approximately 11–20 μM, indicating both high affinity for the substrate and high antitumor activity [22,25,26].

The antitumor properties of L-asparaginase are due to its hydrolytic capacity. This enzyme belongs to the group of amidase enzymes and can decompose the amino acid L-asparagine into aspartate and ammonia, and also presents glutaminase activity [27]. Asparagine is a non-essential amino acid necessary for protein synthesis and normal cell growth [28].

Its biosynthesis takes place through the conversion of oxaloacetate to aspartate, by the action of the enzyme transaminase, followed by the transfer of the amino group from glutamate to oxaloacetate, generating α-ketoglutarate and aspartate [29]. Healthy cells can synthesize asparagine from the aspartate generated, through the enzyme asparagine synthetase and using ATP as an energy source. In contrast, neoplastic cells depend on the exogenous supply of asparagine for their existence and reproduction, since they do not possess the intracellular capacity necessary to synthesize sufficient amounts [28,30]. Taking this as a basis, the use of L-asparaginase in chemotherapies was implemented and it was shown that in tumor cells the hydrolysis of asparagine by L-asparaginase drains all circulating asparagine, resulting in the depletion of serum asparagine [28], leading to cancer cell starvation and ultimately to DNA breaks, cell cycle arrest, and apoptosis. The molecular mechanism by which ASNase induces asparagine and glutamine depletion is linked to inhibition of the mTOR pathway. The inhibition of mTOR causes the inhibition of downstream events in its signaling cascade, such as the phosphorylation of serine threonine protein kinase (p70S6 kinase or p70s6k) and eukaryotic initiation factor 4E-binding protein 1 (4E-BP1), thus suppressing ribosomal protein synthesis. Additionally, it has been demonstrated that the action of ASNase triggers a series of events such as an increase in caspase-3, the release of cytochrome c, the activation of poly (ADP-ribose) polymerase, a decrease in the anti-apoptotic protein Bcl-XL, activation of the GCN2 pathway, cell cycle arrest in the G0/G1 phase, and eventually, apoptosis [29,31]. All of the above induce a reduced viability of leukemic cells.

As a result of these observations, therapies containing L-asparaginase have been developed for the treatment of hematological malignancies, including acute lymphoblastic leukemia (ALL).

### 1.2. Main Adverse Responses to L-Asparaginase (ASNase) Treatment

All therapeutic proteins have the potential to induce anti-drug antibody (ADA) production. The L-asparaginases in clinical use are a notable example of a well-established therapeutic protein with the ability to elicit an immune response in patients, which is due, in part, to its bacterial origin and large size [32]. Problems associated with immunodeficiency and acute liver dysfunction are the main side effects of ASNase in leukemia therapy [33]. When L-asparaginase is administered to the human body, normal and leukemic lymphoblasts can cleave it via the lysosomal proteases cathepsin B and asparagine endopeptidase [34], enhancing antigen processing and promoting an immune response [35,36]. This immune response is the main cause for the discontinuation of L-asparaginase treatment and is known as hypersensitivity [4]. Hypersensitivity reactions as an adverse effect are well described [5,37], due both to the production of high titers of IgG3 [38] and IgE [39] antibodies and to the adverse effects this causes.

Adverse reactions induced by hypersensitivity include anaphylaxis, edema, serum sickness, bronchospasm, urticaria and exanthema, pruritus and swelling of the extremities, and erythema [12,40]. Some reports indicate that *E. coli* ASNase causes more hypersensitivity reactions than that derived from *Erwinia* sp. with rates ranging from 20 to 30% in patients treated with native *E. coli* ASNase [41,42].

Anti-L-asparaginase antibodies affect the drug’s pharmacokinetics and clearance, shortening its half-life [43], reducing circulating enzyme activity [44], and causing antigen–antibody complex deposition in the mononuclear phagocytic system [45], leading to decreased drug potency. This results in subclinical hypersensitivity or silent inactivation, where the drug’s efficacy is lost but the patient remains asymptomatic, not exhibiting any signs of an adverse reaction despite the drug inactivation, causing poor outcomes [46]. Cross-reactivity of antibodies against *E. coli* ASNase has also been seen in patients treated with *Erwinia*-derived or PEGylated *E. coli* ASNase, though the cause is unclear [47].

In general, this is a major disadvantage of the use of bacterial enzymes as therapeutic agents.

### 1.3. Approaches to Reduce the Immunogenicity of L-Asparaginase

Several strategies have been developed to reduce the immunogenicity of ASNase and prolong its half-life in the blood, which would allow for more widely spaced dosing intervals. One of the solutions that have been found for immunogenicity against this enzyme has been PEGylation, a process by which the enzyme is covalently coupled to the polymer polyethylene glycol. Once L-asparaginase is conjugated with PEG, the exposed positions on the surface that are not involved in the binding site are covered, increasing the molecular size, preventing the uptake of L-asparaginase by the reticuloendothelial system, and decreasing the probability of generating ADA [48,49]. In this case, the most recent available on the market is PEG (Oncaspar) or succinimidyl carbonate (SC) PEG (Calaspargase pegol) with native EcA [48,49]. PEGylated L-asparaginases are now used in first-line therapy in some countries and are rapidly becoming the preferred preparation because of their lower degree of immunogenicity [50]. Although PEGylation of this enzyme has improved its therapeutic efficacy, this process produces alterations in its activity and kinetic parameters [51]. There is research reporting side effects and silent inactivation for the PEgylated enzyme [52,53]. Many patients have frequently shown significant cross-resistance between these formulations [54], and there are also reports of non-fatal toxic adverse effects, especially hepatotoxicity, mainly in adults older than 39 years [55].

N-glycosylation is a posttranslational modification exploited for the expression of recombinant “humanized” proteins of therapeutic interest, using yeast (*Pichia pastoris*) as the expression system [56]. Thus, N-glycosylation is a potential strategy that could minimize the immunogenicity of ASNase. In 2019, Effer et al. obtained ASNase from *Erwinia chrysanthemi* with different levels of N-glycosylation [57], using GlycoSwitch^®^ yeast strains, genetically modified for the production of recombinant proteins with a homogeneous N-glycosylation pattern similar to humans [58,59]. This mutant represents an excellent alternative for the recombinant production of low-immunogenic ASNase for the treatment of ALL.

Another strategy used to decrease ASNase immunogenicity is site-directed mutagenesis. Early studies date back to Moola in 1994, who used mouse and rabbit anti-ASNase antibodies from *Erwinia* sp. produced against 10 continuous hexapeptides to map the epitope positions in this enzyme and found that most immune responses were generated against peptide 282–292, so he replaced each residue with the other 19 natural amino acids [60].

In general, he found that most mutations reduce immunogenicity, especially those that cause a change in charge, aromaticity, or size and reported that Pro291 is of particular importance [60]. With the emergence of bioinformatics, scientific technological development has been revolutionized by applying computational technologies to the management and analysis of biological data. Using protein databases such as the Protein Data Bank (PDB) and bioinformatics tools such as NetMHC II and PyMol, it has been possible to predict the immunogenic epitopes of many proteins and their location in the three-dimensional structure of proteins. This makes bioinformatics a powerful and necessary tool to incorporate in studies related to the de-immunogenization of proteins that have applicability as biopharmaceuticals [33,41,61]. Site-directed mutagenesis studies using these tools have shown that the de-immunogenization of therapeutic proteins by modifying bioinformatically predicted T cell or B cell epitopes is a successful strategy for obtaining safer therapeutic products. Since the *E. coli* enzyme has ahigh immunogenicity and allergenicity, this applicability has been extended to research concerning L-asparaginase, generating by combining in silico and in vitro methods assessing mutants of the *E. coli* ASNase enzyme that are less immunogenic and with potential chemotherapeutic use [62,63,64]. Some studies, on the other hand, have revealed that the placement of three consecutive alanines at position 196 (195-AAA-197) substantially decreases immunogenicity in EcA [65]. However, many attempts have been unsuccessful in conserving ASNase activity or significantly decreasing its immunogenicity by mutagenesis.

Finally, in the GRASPA^®^ formulation, the strategy of encapsulation of the enzyme in erythrocytes was used. The erythrocyte membrane protects ASNase against degradation and elimination and this could have the potential to prolong enzyme activity and reduce toxicity [66]. Other approaches to reduce the risk of antibody formation such as the administration of L-asparaginase with immunosuppressive steroids have been proposed, but also present certain complications [40].

Chimeric proteins have been widely used in studies for therapeutic purposes. In 2004, Ahmi Ben Yehudah et al. developed a targeted cancer therapy with chimeric gonadotropin-releasing hormone (GnRH) proteins [67]. The GnRH-based chimeric proteins designed in the study selectively destroyed adenocarcinoma cells both in vitro and in vivo, which allowed them to conclude that the use of chimeric proteins for targeted therapy represented a novel therapeutic modality for the treatment of cancer in humans [67]. In 2011, Haya Lorberboum-Galski and co-workers developed “Human Killing Moieties” which are nothing but human toxins, based on recombinant immunotoxins and chimeric proteins, as a drug delivery system for the targeted treatment of human diseases [68]. Jong Youl Lee, in 2021, developed a chimeric protein of Exendin-4, a clinically available anti-diabetic peptide with a short plasma half-life, by genetically fusing an albumin-binding domain (ABD) and an anti-neonatal Fc receptor antibody (AFF) [69]. This study increased the half-life of the drug 241-fold, demonstrating that the development of these chimeric proteins could be an effective strategy to greatly increase the plasma half-life of therapeutic proteins and, therefore, significantly improve their pharmacological capacity [69]. Making use of bioinformatics analysis and in silico design, Ting Nie and co-workers, in 2021, developed four novel chimeric lysins (P361, P362, P371, and P372) by fusing Salmonella phage lysins and the antimicrobial peptide LeuA-P [70]. The recombinant chimeric lysins were expressed in the *E. coli* strain BL21 (DE3) and showed highly specific inhibition against Salmonella [70].

Associated with ASNase, previous work involving the production of chimeric proteins has also been developed. Newsted et al., in 1995, demonstrated that the resistance of L-asparaginase to proteolytic degradation by trypsin can be increased by developing a chimera comprising a fusion of the ASNase gene with that of a single-stranded antibody derived from a pre-selected monoclonal antibody capable of providing protection against trypsin [71]. Engineered chimeric L-asparaginase retained 75% of its original activity after exposure to trypsin, whereas the native L-asparaginase control was completely inactivated [71]. In 2006, Qi Gaofu and coworkers developed a chimeric variant comprising asparaginase, the tetanus toxin helper T-cell epitope, and the B-cell epitope of human coresteryl ester transfer protein (CETP). The chimeric enzyme was expressed as a soluble protein in *Escherichia coli* and once purified exhibited approximately 83% native asparaginase activity [72]. Subsequent studies in animal models raised the possibility of using this protein as a vaccine against atherosclerosis. Recently, Belén and coworkers, in 2021, developed in silico a chimera of L-asparaginase by replacing epitope peptides in the enzymatic variant of *E. coli* with peptides from human serum albumin, even demonstrating proof of concept that the designed variant is recombinantly expressed in *E. coli* (paper accepted for publication). In relation to the human enzyme, mutant chimeras of the enzyme and low-KM guinea pig asparaginase have been designed using random DNA mixing, creating human asparaginase variants with a sequence identity of 90%, but with low KM values [50], which reflected the need to contemplate other perspectives in relation to human ASNase [26].

Despite these advances, finding variants of this enzyme with lower immunogenicity for therapeutic use continues to be a challenge. In view of the growing need for new variants of L-asparaginase for therapeutic purposes that are less detectable by the immune system and based on the use of bioinformatics tools for protein deimmunogenization, the present research proposed obtaining a recombinant humanized chimeric enzyme from ASNase from *E. coli* and ASNase from *H. sapiens*. With this variant, a novel, physically and chemically stable enzyme is sought, with optimal kinetic properties and lower immunogenicity than the native variant of *E. coli*, guaranteeing its potential applicability as a biopharmaceutical. This study presents a novel and rational approach to reducing the immunogenicity of bacterial L-asparaginase by combining in silico epitope mapping with targeted structural engineering. Unlike conventional methods such as PEGylation or random mutagenesis, our design replaces predicted antigenic and allergenic surface epitopes in *E. coli* ASNase with homologous peptide fragments from human L-asparaginase. These replacements preserve the secondary structure and avoid the catalytic site, ensuring enzymatic activity is maintained while potentially reducing immune recognition. Although *Pichia pastoris* is commonly used for expressing humanized proteins, we deliberately chose *E. coli* as the expression host to prevent unwanted glycosylation, enabling efficient production of a non-glycosylated, active chimeric enzyme. This combination of rational immunomodulatory design and a simplified, cost-effective prokaryotic expression system highlights the innovation and translational potential of our work.

## 2. Results and Discussion

Human and bacterial L-asparaginases exhibit low sequence similarity (19.84%) due to their distinct evolutionary origins, but they share the function of degrading asparagine thanks to conserved structural residues. Although the human enzyme is not clinically useful because of its low substrate affinity, its human origin reduces the risk of hypersensitivity, making it a suitable model for designing a humanized chimeric version with lower immunogenicity.

In a previous study, the present research team designed a humanized chimeric enzyme by replacing two immunogenic 15-amino-acid peptides from the *E. coli* bacterial variant with human fragments selected for their high affinity to MHC class II molecules, aiming to reduce the immune response. This substitution reduced the number of immunogenic epitopes by 14 compared to the bacterial protein, thereby decreasing the likelihood of hypersensitivity in patients with acute lymphoblastic leukemia. Considering the structural characteristics of the designed chimeric protein, it was observed that the substituting polypeptide chains retain the same alpha-helical secondary structure as the corresponding fragments of the native template protein 3ECA. Furthermore, the three-dimensional structure of the chimeric protein demonstrated that the enzyme preserves the active conformation of the native protein, forming a homotetramer with α/β domains connected by a linker [73]. These data are presented in Supplementary Figures S1–S4.

### 2.1. Obtaining the Recombinant Plasmid with the Inserted Gene of Interest

In the process of obtaining the recombinant humanized chimeric enzyme, the first stage consisted of the amplification of the synthetic gene. For this, the lyophilized gene was used together with the primers acquired through the company Integrated DNA Technologies (IDT) and amplified by Polymerase Chain Reaction (PCR).

**Figure 1:**
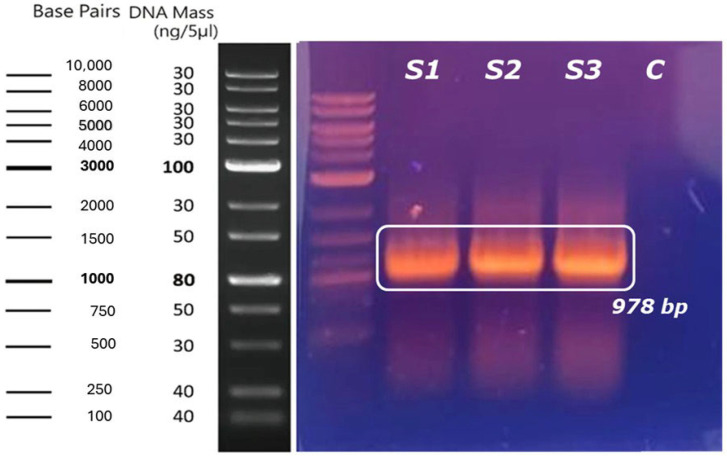
1% agarose gel electrophoresis of the amplification product of the humanized ASNase_Chimeric _*E. coli-H. sapiens* gene. The amplified bands of the gene of interest are indicated within a rectangle. Lanes S1, S2, and S3 are replicates of the sample and show a specific band of approximately 978 bp, corresponding to the expected size of the synthesized gene. Lane C represents the negative control without DNA template. A molecular weight marker (DNA ladder) is shown on the left, with the 1000 bp band used as a reference for estimating the product size.

Figure 1 presents the results obtained after applying 1% agarose gel electrophoresis to the PCR amplified product. In the image, three bands positioned around 1000 base pairs (bp) can be identified, which is consistent with the expected size of the engineered chimeric humanized asparaginase (ASNase) gene (978 bp) and confirms the correct amplification of the DNA fragment of interest. No bands corresponding to the blank control of the reaction were observed, indicating the absence of contaminants and confirming that the observed bands correspond to the gene of interest, which validates the specificity of the amplification process.

An extension temperature of 72 °C was used for optimal activity of Platinum Taq DNA Polymerase High Fidelity (Invitrogen), enabling efficient amplification of the gene of interest with high fidelity and minimizing unwanted mutations [74].

These results indicate that the amplification of the humanized chimeric ASNase gene was successful and was achieved at adequate concentrations, which is in agreement with the results presented by Herrera Belén et al. in 2020 and Effer and in 2019, who using the same PCR methodology obtained asparaginase genes amplified with equal quality and with similar concentrations [57,61]. However, in the present study, faded bands were observed at the bottom of the gel, suggesting nonspecific products or degraded fragments. This phenomenon could be attributed to a suboptimal annealing temperature, resulting in non-specific hybridization of the primers and consequently amplification of additional fragments [75,76,77,78]. This coincides with the results reported by Barboza-Fallas in 2019, who investigated the optimization of the annealing temperature in a multiplex PCR for the detection of Listeria monocytogenes, tested different annealing temperatures, and found that an optimal annealing temperature, in their case 57 °C, provided the highest specificity, avoiding nonspecific amplifications [79].

Although gene purification was not affected, optimizing annealing temperature via a thermal gradient is recommended to minimize nonspecific products and improve amplification efficiency, ensuring higher DNA purity for cloning and expression [79]. Additionally, nonspecific amplification at low temperatures can be reduced using hot-start techniques, where DNA polymerase is added or activated at elevated temperatures to prevent nonspecific primer binding [80].

### 2.2. Digestion and Ligation of the Gene of Interest in the Expression Vector

PCR products were purified using a commercial Gel Extraction Kit (Omega BIO-TEK) to remove contaminants that could affect subsequent digestion with the pET-22b (+) plasmid, improving cloning efficiency [81]. Both the gene and vector were digested with NcoI and XhoI restriction enzymes, which target specific sequences added via primers and the vector’s multiple cloning site, generating cohesive ends to facilitate ligation [82,83,84,85]. Enzymatic digestion was verified by 1% agarose gel electrophoresis, confirming the expected DNA fragments (~1000 bp for the gene and ~5400 bp for the pET-22b (+) plasmid). The undigested control showed supercoiled and relaxed plasmid forms [84,85]. This analysis was considered secondary; therefore, the electrophoresis image was not included. Subsequently, the digested DNA was extracted and purified using the “Gel Extraction Kit” (Omega BIO-TEK). This was a key procedure to eliminate reagent and enzyme residues that could interfere with the subsequent ligation reaction. This step is fundamental, since contaminants such as salts, undigested DNA fragments, and enzyme residues can reduce the efficiency of the ligase and affect the correct insertion of the gene into the expression plasmid [86].

Binding of the gene of interest to the pET-22b (+) vector was performed by a ligation reaction using the enzyme T4 DNA ligase, following the standard protocol described by Sambrook & Russell (2001) [87,88]. The construction of the recombinant plasmid included a sequence of six histidines (His-tag) at the N-terminal end of the fusion protein, for efficient purification of the recombinant protein at later stages by affinity chromatography techniques, facilitating biochemical and functional studies of the chimeric enzyme.

Efficient digestion and purification of the gene and pET-22b (+) vector ensured high-quality material for ligation, a crucial step in generating the recombinant construct. However, low product concentration after ligation prevented verification by agarose gel electrophoresis, a common issue due to material loss during digestion and purification [85,87]. To optimize plasmid transformation, a dialysis step was performed to remove salts and contaminants that could reduce transformation efficiency [86].

### 2.3. Transformation

The modified genetic construct was introduced into electrocompetent DH5-α cells via electroporation, followed by selection on antibiotic-supplemented LB agar to isolate clones carrying the plasmid [89]. Of five clones, three showed favorable growth with well-defined colonies, indicating successful transformation and antibiotic resistance. Variation in colony growth may result from differences in transformation efficiency, plasmid quality, electrical pulse conditions, or possible plasmid rearrangements [85,90].

**Figure 2:**
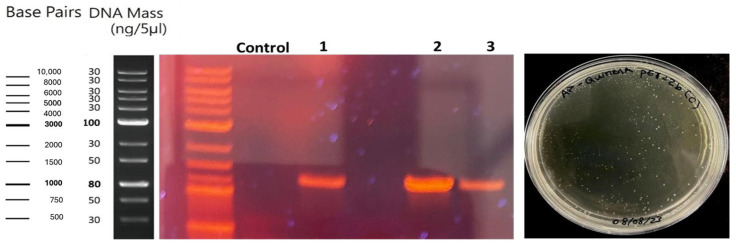
Electrophoresis in 1% agarose gel of the PCR reaction of the colonies transformed with the recombinant plasmid containing the gene of interest. Bands are observed at a height of approximately 1000 bp corresponding to the plasmid transformed and amplified by the presence of the gene. Nothing is observed in the control or blank.

The selected colonies were subjected to colony PCR (colony PCR) in order to amplify the plasmid of interest and verify the presence of the gene inserted in the pET-22b (+) vector. Figure 2 shows the results of the amplification of the three recombinant clones, where the presence of bands around 1000 bp is observed, which is in agreement with the expected length of the amplified fragment. These results suggest that the selected cells successfully integrated the modified vector with the chimeric enzyme gene.

These bands would only form if the bacteria correctly incorporated the recombinant plasmid. No nonspecific amplifications or additional fragments were detected, indicating an efficient insertion with no detectable rearrangements at this stage.

#### Transformation of BL21 and Rossetta Strains and Chimeric Protein Expression Assays of Chimeric ASNase Chimeric Protein in *E. coli* BL21 and Rosetta

*E. coli* was chosen as the host for recombinant enzyme expression due to its fast growth, ease of genetic manipulation, low cost, variety of stable vectors, and regulatory approval for biopharmaceutical production [91,92]. The strains BL21 (DE3) and Rosetta (DE3) were used for expressing chimeric ASNase. Selected clones were transformed by electroporation and cultured on antibiotic-supplemented LB plates to select colonies with adequate growth. Three Rosetta clones (C2, C4, C5) and two BL21 clones (C2, C4) were chosen for expression assays [93,94]. Pre-inoculations were grown with appropriate antibiotics, induced with 1 mM IPTG at OD 0.6–0.8, and cells were harvested and lysed using BugBuster (Merck Millipore, Darmstadt, Germany). Expression of the chimeric protein was analyzed by SDS-PAGE from the soluble and insoluble fractions.

**Figure 3:**
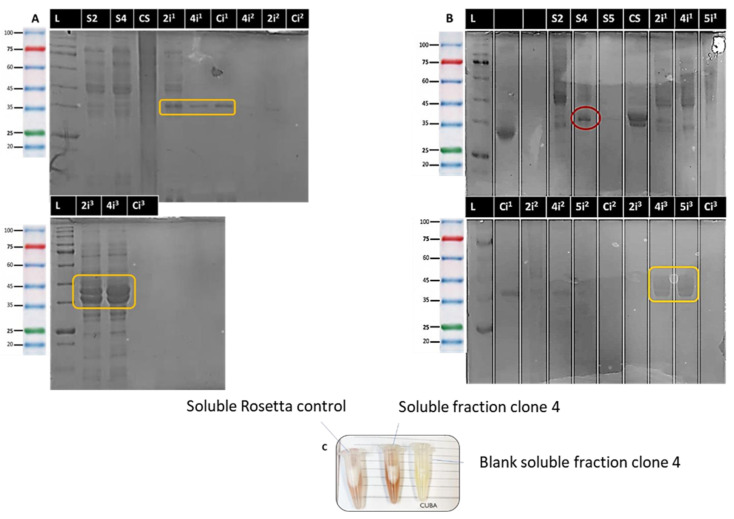
Humanized_*E. coli-H. sapiens* chimeric ASNase enzyme expression and activity assays after the induction process. (**A**) SDS-PAGE electrophoresis at 14% of the soluble(s) and insoluble fraction (i1, i2, and i3), and control for both fractions (Cs and Ci) for clones 2 and 4, using the *E. coli* BL21 strain as a host. (**B**) SDS-PAGE electrophoresis at 14% of the soluble(s) and insoluble fraction (i1, i2, and i3), and control for both fractions (Cs and Ci) for clones 2, 4, and 5, using the *E. coli* Rosetta (DE3) strain as a host. (**C**) Qualitative hydroxylamine enzyme activity assay of the soluble and insoluble fraction (i1, i2, and i3) using the *E. coli* Rosetta (DE3) strain as a host, *E. coli* Rosetta (DE3) strains modified with the empty vector as a control, and substrate without enzyme as the blank reagent. The brown coloration indicates L-asparaginase activity through the formation of a complex with hydroxylamine.

The results obtained, presented in Figure 3, showed significant differences in expression between the BL21 and Rosetta strains. In BL21 (Figure 3A), few defined bands were initially observed, so the experiment was repeated. In this second evaluation, more defined bands were identified around 35 kDa, the approximate molecular weight of the protein of interest, especially in insoluble fraction 1 and, more intensely, in insoluble fraction 3, suggesting the formation of inclusion bodies [95,96].

On the other hand, in the strain Rosetta (Figure 3B), bands corresponding to the chimeric protein were clearly visible in insoluble fractions 1 and 3, indicating an expression profile similar to that of BL21. However, unlike BL21, a band corresponding to the expected molecular weight was detected in the soluble fraction of clone 4, suggesting a higher proportion of protein in soluble and possibly functional conformation in this strain.

To complement these findings, qualitative enzyme activity assays were performed using hydroxylamine. In BL21, no differences in staining were observed between the soluble and insoluble fractions of the modified clones and the control construct (empty vector), which contrasts with the results obtained in SDS-PAGE electrophoresis. This suggests that, although the chimeric protein was overexpressed, it is likely to be in a nonfunctional conformation, possibly due to its accumulation in inclusion bodies [96]. This situation could be attributed to mutations during transformation in DH5-α cells [97,98,99], as well as to the particular nature of the gene sequence of interest. This sequence is unusual in that it incorporates human DNA fragments. Furthermore, it is plausible that this circumstance is related to a possible incorrect folding of the three-dimensional structure of the protein. As a result, the expression and export of the enzyme to the periplasm could be being affected [100].

In contrast, in Rosetta (Figure 3C), qualitative assays of enzyme activity evidenced a more intense staining in clone 4, consistent with the presence of the protein in the soluble fraction observed in SDS-PAGE. In this case, the control construct did not show a characteristic chromogenic reaction, confirming that the staining observed in clone 4 is associated with the specific enzymatic activity of the chimeric ASNase. In many cases, the expression of the enzyme can occur, but it could be due to endogenous ASNase, since *E. coli* is capable of producing its own ASNase, which would give us a false positive result, so it would be advisable to silence the genes of this intrinsic protein [101]. In the present assay, taking this as a basis, the qualitative analysis was also performed for an empty vector, which ruled out that the staining obtained was due to the activity of the endogenous enzyme. This showed a lower coloration than that obtained for clone 4.

The result obtained was corroborated by the Nessler method, which allowed us to demonstrate that clone 4 of the Rosetta strain was indeed expressing the designed chimeric protein, reaching an enzymatic activity of 1.98 U/mL. This value confirms the expression and functionality of the humanized chimeric protein, although it still remains below the levels observed for the enzyme in its native form. The Rosetta strain has been successfully used for the expression of recombinant proteins from a variety of sources, including human and viral proteins [102,103,104,105]. This strain has been genetically modified and has a number of expression codons that are considered rare, which contributes to the improved expression of proteins of eukaryotic origin. In addition to this, there is the possibility that the proteins manage to acquire a proper folded structure in the cytosolic fraction. This is due to the mutation of several enzymes involved in the folding process [106,107]. This background may explain the fact that the protein is being expressed in this strain and not in BL21. Similar studies have been carried out by other researchers, obtaining similar results to the present experiment in both strains. Such is the case of the research led by Tegel in 2010, where a comparison of the production of human protein fragments was performed using the *E. coli* BL21 (DE3) and *E. coli* Rosetta (DE3) strains. It was found that the Rosetta (DE3) strains exhibited increased yield and higher purity for more than 11,000 expressed proteins, indicating a higher success rate in the process and greater affinity of the transcriptomic machinery of this strain for the translation and expression of rare codons, especially of humanized proteins [108]. There is also the study performed by Caetano, L.F. (2020), who focused on the production and preliminary characterization of *E. coli* L-asparaginase II variants of lower immunogenic potential and combined this with in silico and in vitro studies. This showed that one of the most feasible strains to use is the Rosetta strain [109].

Despite these results, it is advisable to modify both the primers and the plasmid in question in subsequent assays, since it may be the case that the histidine tail and the rest of the construct are carrying too much weight for the cellular machinery of both strains to express the protein in a feasible manner and in greater quantities [109]. Another factor that could be restricting the efficient expression of L-ASNase is the possible presence of a suboptimal signal peptide. To facilitate release and obtain a soluble L-ASNase in the periplasm, the signal peptide pelB was incorporated into the design of this enzyme. The choice of an inappropriate signal peptide could affect the efficiency of enzyme production, as it could result in the accumulation of the enzyme in incorrect compartments or incorrect translocation. One way to address this problem is through signal peptide modification. Native signal peptides can be replaced by more efficient and previously validated ones, as has been demonstrated in previous research [110].

### 2.4. Optimization

**Figure 4:**
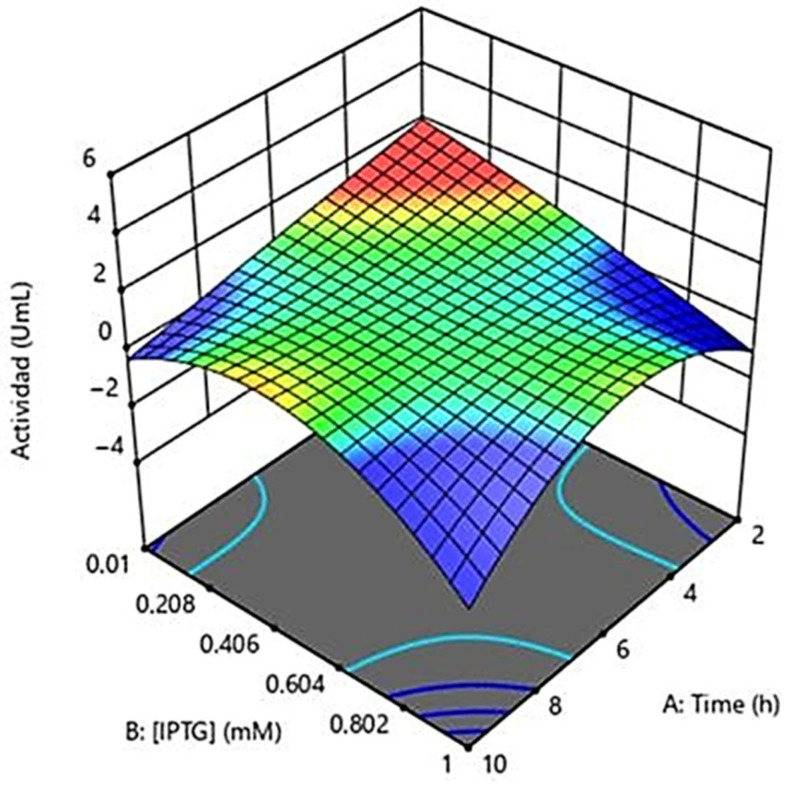
Response-surface plot representing the behavior of enzymatic activity in relation to IPTG concentration (mM), time (hours), and temperature (°C), generated using Design-Expert software. This software fixes one variable at a constant value while displaying the effects of the other two variables. In the presented graph, the Z-axis corresponds to enzymatic activity, while the X and Y axes represent IPTG concentration (mM) and time (hours), respectively. For this case, the temperature was fixed at 37 °C. The optimal conditions observed were an IPTG concentration of 0.01 mM and a time range of 2 to 4 h, where a maximum activity of approximately 2 U/mL is indicated in red on the plot. These values represent the optimal conditions for enzyme production. The data were fitted to a quadratic model, and ANOVA analysis indicated no significant differences (*p* > 0.05), confirming the adequacy of the model.

A response-surface experimental design was used to optimize IPTG-induced enzyme expression by evaluating the temperature, IPTG concentration, and induction time [111]. Four levels for each variable were tested to identify optimal conditions. The best enzymatic activity of the chimeric protein was achieved at 37 °C, 0.01 mM IPTG, and 2–4 h induction. Statistical analysis showed no significant differences (*p* > 0.05), validating the experimental model Figure 4.

These results are in contrast to studies by Lefin in 2023 (in edit) and Effer in 2019 [57], with the main differences between the two experimental approaches being the concentration of IPTG employed and the induction time. Whereas Lefin et al. employed a concentration of 1 mM, the present study determined that a significantly lower concentration of 0.01 mM is optimal for chimeric enzyme expression. This discrepancy could be attributed to variations in the genetic constructs, the *E. coli* strains used, or the specific characteristics of the chimeric protein in question [57,103,112].

The scientific literature supports the idea that lower concentrations of IPTG can be effective and even beneficial for the expression of recombinant proteins in *E. coli*. For example, studies have shown that reduced concentrations of IPTG can minimize the formation of inclusion bodies and improve the solubility of the expressed protein [113,114]. In addition, the use of lower concentrations of inducer can reduce the metabolic burden on host cells, favoring more efficient expression of the protein of interest [113,114,115].

Regarding the time variable, in the present study it was determined that a time of 2 to 4 h is the optimal time to obtain a good expression of the chimeric enzyme; on the other hand, previous studies such as those carried out by Effer and Munhoz refer to 4 h as the optimal condition of this variable to obtain significant results [116]. It is known that the induction period is crucial to allow adequate protein expression without compromising cell viability [115]. Longer induction times may lead to the accumulation of misfolded proteins or proteolytic degradation, while too-short times may result in insufficient production of the protein of interest [117]. This time interval balances maximal production of the chimeric enzyme with protein functionality and stability, aligning with studies suggesting that induction duration should be carefully optimized for each expression system.

As a result of this assay, an activity of 2.4 U/mL was obtained, indicating an increase in asparaginase activity with respect to the initial measurement by Nessler. This increase in activity over previous assays can be attributed to the optimization of factors related to the regulation of expression and proper folding of the chimeric protein [113]. The optimization of these factors favored more controlled expression and solubilization of the protein, allowing more of the enzyme to remain in its active form. Additionally, they contributed to the optimal activity of the cellular chaperones responsible for protein folding and avoided negative effects such as the depletion of cellular resources, accumulation of toxic products, and degradation of the recombinant protein, which ensured a greater production of active enzyme.

The response-surface model shows a positive interaction between temperature, low IPTG concentration, and induction time, emphasizing the need for precise control to maximize enzyme expression and activity. The low IPTG concentration required suggests cost-effectiveness for scale-up. The standard 37 °C temperature and 2–4 h induction provide operational flexibility. This model offers a robust basis for future optimization to efficiently enhance chimeric enzyme activity.

### 2.5. Culture Scale-Up and Purification

The culture of clone 4 from Rosetta was scaled up to 1 L in medium containing ampicillin and chloramphenicol, induced with IPTG, according to the optimized conditions, when the OD reached between 0.6 and 0.8, and subsequently centrifuged. The pellet was lysed by buffer and sonication to evaluate periplasmic expression of chimeric ASNase. The cell-free extract was filtered and purified by nickel-affinity chromatography using HisTrap™ HP columns on an AKTA system, with buffer A (50 mM sodium phosphate, 300 mM NaCl, 100 mM imidazole, pH 7.4). An aliquot before purification was taken for efficiency analysis. Proteins were eluted with an imidazole gradient (100–500 mM).

**Figure 5:**
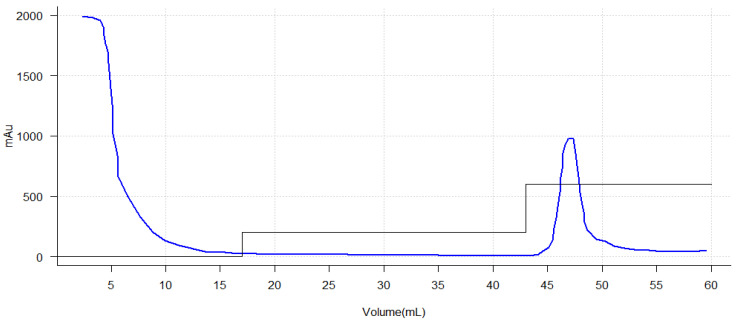
Chromatogram of ASNase_H_Q protein purification by histidine-tailed affinity chromatography using a nickel column on the AKTA Star. On the x-axis, in the ml column the volume is represented, and on the y-axis the absorbance (mAu) is represented. A peak between 44 and 50 mL, corresponding to the elution of the target protein, is observed. Elution was performed using a stepwise imidazole gradient from 100 to 500 mM; therefore, each step represents a specific concentration within the gradient. The protein eluted at 30%, corresponding to 150 mM imidazole.

The purification of histidine-tagged recombinant proteins by immobilized metal affinity chromatography (IMAC) is a widely used technique due to its efficiency and specificity. As can be seen in Figure 5, a graph showing the result of the purification performed can be seen. In the study performed, a characteristic chromatographic profile was observed during the purification process of the chimeric enzyme. In the initial phase (0–20 mL) a high absorbance was observed (about 2000 mAU at the beginning), which corresponds to the initial loading of the sample on the column. The rapid decrease in absorbance indicates that non-specific components or contaminants in the sample are being removed during the initial wash phases. In the 20–40 mL phase, elution buffer was applied which would remove weakly bound proteins from the affinity column. The absorbance remains close to zero, suggesting that the wash was effective in removing non-specific contaminants and that in that elution range there are no proteins bound to the matrix. For the 40–50 mL elution phase, starting at 45 mL a significant increase in absorbance was observed, peaking around 47–48 mL with a height of 1100 mAU and a concentration of 150 mM imidazole. This indicates elution of the protein of interest, due to the application of the imidazole gradient. The height and shape of the peak suggests concentrated elution of the histidine-labeled protein. In the final phase, greater than 50 mL, after the peak, the absorbance returns to low values, indicating that the protein of interest has been completely eluted.

The main peak around 47 mL is indicative of the histidine-tagged recombinant protein, which binds specifically to the affinity resin. The absence of additional peaks suggests good specificity of the purification system, with minimal co-eluted contaminations. The intensity and area under the peak provide a qualitative estimate of the amount of purified protein.

These findings are consistent with previous studies such as Meena’s 2016 research, in which purification of recombinant L-asparaginase was carried out using a Ni-NTA affinity column [118]. In the initial loading phase, both studies showed high absorbance, which is expected given the mixture of proteins and contaminants present in the crude extracts. During the wash phase, a buffer was used that kept the absorbances close to zero, indicating an effective and successful wash in removing unwanted proteins. In the elution phase, Meena’s study reported an increase in absorbance after imidazole application, which allowed identification of the purified protein concentration, similar to the present study, reflecting a clear elution of the protein of interest. Upon completion, in agreement with the present study, there was a need to check the absorbance, which returned to low values, confirming that the protein of interest had been completely eluted [118]. However, the Meena study presented additional challenges with contaminants in the eluted fractions, whereas the present analysis demonstrated an absence of additional peaks, suggesting a higher purity in the fractions and an optimized purification protocol. This is indicative that a more homogeneous purification with fewer contaminants was obtained in the present investigation, thus highlighting the relevance of adjusting the experimental conditions in the purification processes.

The results presented are also in correspondence with those presented by Effer and collaborators in 2019, who used the same technique and the same methodology, differing only in the elution point since they presented an elution in a percentage higher than 30% of the imidazole gradient [57]. This may be due to the difference in the affinity of the protein for the matrix and in turn for the type of protein, since the protein under study is a humanized chimera and the one investigated by Effer was a glycosylated asparaginase. This may induce variations in the degree of folding and thus exposure of the histidine tail, which mediates the binding and affinity of the protein for the column matrix during the purification process.

Other investigations such as the one carried out by Faizan Muneer [119] obtained similar results in purification, highlighting the effectiveness of L-asparaginase purification techniques. As in the present study, the importance of an effective washing is pointed out, which is evidenced in the absence of contaminants after washing. Although ion-exchange and gel-filtration methods were employed to improve the specificity and yield in the recovery of L-asparaginase, the elution step also showed the concentrated elution of the labeled protein [119]. Also, the absence of additional peaks after elution supported the article’s conclusion on the high purity of the enzyme obtained, confirming the efficiency of the purification system used in the present experiment [119].

**Figure 6:**
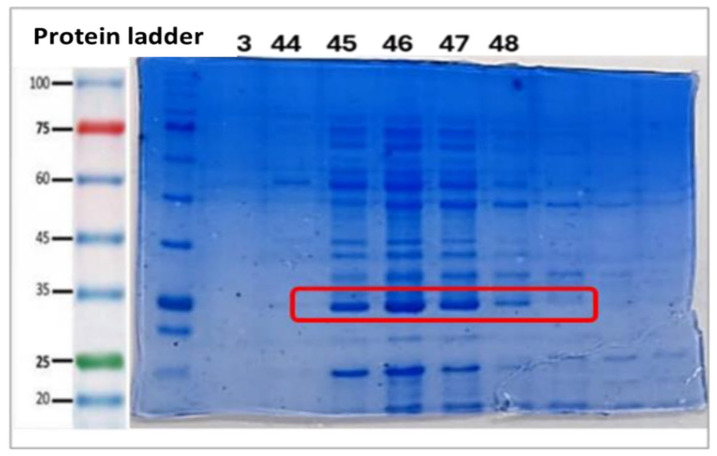
12% SDS-PAGE electrophoresis of the soluble fractions collect of the chromatography. The protein of interest in the 35 kDa range is shown in red.

To evaluate the efficiency of the chromatography, two analyses were performed: first, polyacrylamide gel electrophoresis (SDS-PAGE) (Figure 6) to visualize the purified proteins; second, a protein concentration measurement by Nanodrop, comparing the purified fractions with a previous aliquot to determine the yield of purified protein.

The protein concentration obtained after purification (2.8 mg/mL) was found to be close to the initial concentration (3 mg/mL), indicating excellent retention of the protein of interest. In standard expression systems (*E. coli*), recombinant protein concentrations in the total lysate usually range from 1 to 10 mg/mL [120], depending on expression efficiency, so results of 3 mg/mL fall within this range. After purification, concentrations are usually lower due to the removal of contaminating proteins, which is expected. In terms of purity, the most defined and cleanest bands in the fractions were those between 45 and 47, as seen in Figure 6, suggesting that these contain the purified protein with a good degree of purity. These results are in correspondence with those obtained by Effer in 2019 and collaborators, Munhoz and collaborators in 2022, and Pourhossein and collaborators in 2014 [116,121], who performed studies on asparaginase and obtained bands close to 35 kDa, which is the weight reported for the monomer of the *E. coli* variant of the native enzyme. In spite of this, it is valid to point out that in many cases the expression of the enzyme can occur, but it could be due to endogenous ASNase, so this electrophoresis partially validates the presence of the designed enzyme.

**Figure 7:**
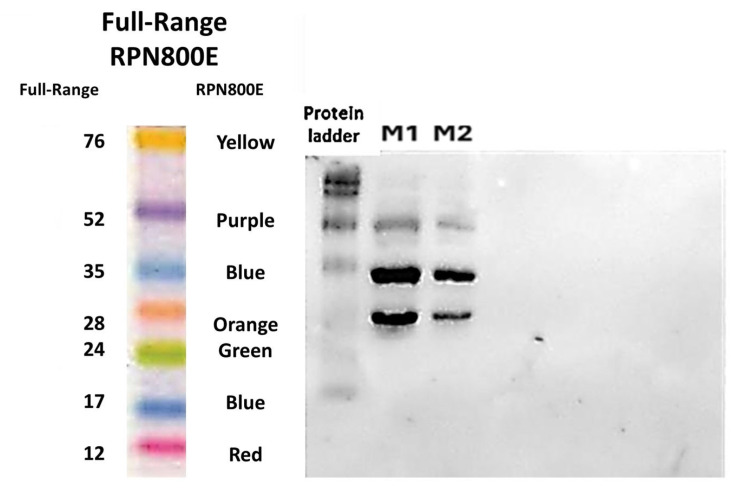
Western blot of ASNASA_H_Q chimeric protein. The black bands below M1 (sample 1) and M2 sample 2 represent the bands of the protein of interest run on electrophoresis and revealed with anti-His antibody from rabbit.

In support of the results obtained, Western blot assays show well-defined bands indicative of the presence of the purified protein, as shown in Figure 7. Additionally, bands close to 28 kDa and 52 kDa, respectively, are seen. In this case, the presence of these bands in the assay is indicative that the histidine tail is present in all three fragments and, therefore, the protein. In the case of the fragment near 38 kDa, it corresponds to the normal protein. The fragments corresponding to 28 kDa may be due to protein cleavage since *E. coli* bacteria present proteases such as metalloproteases, serine proteases, cysteine proteases, and aspartoproteases [122,123,124,125]. Many of these proteases are present in the periplasm together with the protein of interest and are also exposed when sonicating the sample to extract the protein; the interaction of these proteases with the designed protein can induce the proteolysis of protein fragments, decreasing the molecular size of the designed protein but not affecting the histidine tag in this case.

This can be seen in the Western blot as a labeled protein, but of a smaller size than expected, which fits the 28 kDa. In the case of the 52 kDa protein, it may be the fact that a homodimer of the cleaved protein is being formed. A dimer is a macromolecular complex formed by two macromolecules joined noncovalently, such as proteins [126]. Homodimers are formed by the covalent binding of the same protein, in this case two 28 kDa proteins that dimerize can give a homodimer of weight close to that observed in the 52kDa, and by not losing the histidine tail would be observable in the Western blot. This fact justifies the band observed at that weight.

These results are in correspondence with those obtained by the research team of Pawel Strzelczyk in 2023, who evaluated the formation of the L-asparaginase YpAI dimer in the context of its catalytic activity [127]. The study showed that YpAI, despite being typically described as a homotetramer, can function effectively as a dimer and it was established by various biophysical techniques that the dimer is fully active [127].

Although as a preliminary assay the results were promising, it was determined to perform the procedure again, to eliminate impurities and obtain a purer product since it would be used in subsequent characterization assays and in cell lines, which implies greater purity and quantity.

## 3. Materials and Methods

### 3.1. Preparation of Electrocompetent E. coli Cells (DH5α, BL21, Rosetta)

For the preparation of electrocompetent cells, 30 mL of SOB medium was inoculated with a single colony from a fresh plate and incubated overnight at 37 °C. Subsequently, 10 mL of this pre-culture was inoculated into 1 L of SOB medium (1:100 ratio) and cultured at 37 °C with shaking at 200 rpm until an optical density at 600 nm (DO_6000_) of approximately 0.5–0.6 was reached, which took 3 to 4 h. From this point on, all manipulations were performed on ice or at 4 °C. Cells were collected by centrifugation at 1800× *g* for 20 min at 4 °C. The cell pellet was washed with one volume of sterile 10% glycerol. This procedure was repeated twice, varying the volumes of glycerol in each wash: first with 0.5 volume and then with 0.1 volume of sterile 10% glycerol. After each wash, the cells were centrifuged under the same conditions. Finally, the cell pellet was resuspended in 0.5–1 mL of sterile 10% glycerol (calculated according to the initial culture volume). Aliquots of 100 µL were prepared in 1.5 mL Eppendorf tubes and stored at −80 °C for later use.

### 3.2. Obtaining the Gene of Interest

The gene of interest was obtained based on previous research developed by the present team in which a chimeric enzyme based on the L-asparaginases of *E. coli* and *Homo sapiens* was designed in silico. Immunogenic and allergenic epitopes were predicted and evaluated, sequences were aligned to humanize the protein, and its three-dimensional structure was modeled by homology and prediction with AlphaFold. Finally, molecular docking was performed to determine the affinity for the substrate, validating key interactions [73]. This gene was synthesized using the Fermelo platform, together with the corresponding primers.

### 3.3. Amplification of the Gene of Interest

Amplification of the synthetic gene was carried out by Polymerase Chain Reaction (PCR) using previously designed primers. To prepare the primers, they were resuspended at a concentration of 100 µM, and then a PCR primer mix was prepared by mixing 12.5 µL of each primer in 475 µL of molecular biology (BM)-grade water, obtaining a final volume of 500 µL [57]. For the preparation of the dNTPs mix, 10 µL of each dNTP was added in 460 µL of BM-grade water, obtaining a final concentration of 10 µM. Platinum Taq DNA Polymerase High Fidelity (Invitrogen, Thermo Fisher Scientific, Carlsbad, CA, USA) was used as the amplification enzyme, due to its high fidelity and efficiency in the amplification of DNA sequences [57]. Finally, the master mix for the PCR reaction was prepared following the proportions described in Table S1.

PCR amplifications were performed under the following thermal cycling conditions: an initial denaturation at 98 °C for 1 min; followed by 30 cycles of denaturation at 98 °C for 30 s, annealing at 60 °C for 1 min, and extension at 72 °C for 1 min; with a final extension at 72 °C for 10 min. The reactions were subsequently held at 4 °C indefinitely.

After amplification, PCR products were analyzed by agarose gel electrophoresis. The electrophoretic run was performed on a 0.8% agarose gel prepared with 1× TAE buffer (40 mM Tris base, 20 mM acetic acid, 0.05 mM EDTA, pH 8) [57]. Electrophoresis was carried out at 90 V for 50 min. For the visualization of DNA bands, 1.5 µL of ethidium bromide was added to the gel before polymerization. Detection was performed by illumination with ultraviolet (UV) light. MaestroGen 1Kb Plus DNA Ladder [57,128] was used as a molecular weight marker. The bands corresponding to the amplification product were recovered and purified using the commercial kit “Gel Extraction Kit” (Omega BIO-TEK, Norcross, GA, USA), following the manufacturer’s instructions.

### 3.4. Plasmid and Insert Digestion

For enzymatic digestion, 1 µg of each gene was taken in separate Eppendorf tubes and treated with NcoI and XhoI endonucleases. These reactions were performed in triplicate with the aim of increasing insert recovery, due to the low recovery efficiency in gel purification kits. Each reaction was brought to a final volume of 50 µL and incubated at 37 °C for 3 h. Subsequently, to stop the enzymatic reaction, the temperature was raised to 80 °C for 20 min. To verify the correct digestion of the plasmid and insert, double-comb agarose gel electrophoresis was performed. The run was performed on a 0.8% agarose gel in 1× TAE buffer (40 mM Tris base, 20 mM acetic acid, 0.05 mM EDTA, pH 8) at 90 V for 40 min. Visualization of DNA bands was performed by adding 1.5 µL of ethidium bromide, and detection was performed under ultraviolet (UV) light. MaestroGen 1Kb Plus DNA Ladder was used as a molecular weight marker. The bands corresponding to the digested plasmid and insert were purified using the commercial “Gel Extraction Kit” (Omega BIO-TEK), following the manufacturer’s instructions.

### 3.5. Plasmid and Insert Ligation

The digested and purified fragments were subjected to a ligation process using T4 DNA ligase, following the manufacturer’s recommendations. To optimize ligation efficiency, the following equation was used, considering 100 ng of vector as a reference for the insert ratio.

$$\frac{\mathrm{ng}\;\mathrm{of}\;\mathrm{vector}\times \mathrm{insert}\;\mathrm{size}\;(\mathrm{kb})}{\mathrm{vector}\;\mathrm{size}\;(\mathrm{kb})}\times \left(\mathrm{Molar}\;\mathrm{ratio}\frac{\mathrm{insert}}{\mathrm{vector}}\right)=\mathrm{ng}\;\mathrm{of}\;\mathrm{insert}$$

For the ligation reaction, 100 ng of the gene of interest and 100 ng of the pET-22 plasmid were used, which were mixed and adjusted to a final volume of 20 µL. As a control, an additional ligation was performed with the empty pET-22 plasmid. The samples were incubated at 25 °C for 3 h, followed by an enzymatic inactivation process by heating at 70 °C for 10 min. Subsequently, the ligation product was subjected to a dialysis process for salt removal using a 0.25 µm filter for 10–15 min.

### 3.6. Transformation of E. coli (DH5α, BL21, Rosetta) Electrocompetent

For transformation, the electroporator was set at 2.5 kV with a 0.5 ms pulse. Then, 40 µL of electrocompetent cells was removed and kept in a Minicooler. Simultaneously, the plasmid was brought into the laminar flow chamber. The bacteria were thawed for a few minutes and mixed with 10 µL of the plasmid. The mixture was kept in the Minicooler for 1 min. Subsequently, the bacteria were transferred to an electroporation cuvette and subjected to an electric shock. Immediately thereafter, 1000 µL of LB medium without antibiotic was added and mixed with the electroporated bacteria. The suspension was incubated at 37 °C with shaking at 200 rpm for 1 h to allow expression of the resistance gene. During incubation, Petri dishes were prepared by adding 20 mL of LB medium and the corresponding antibiotic according to the resistance conferred by the plasmid, in this case ampicillin, to each one. In this experiment, a plasmid with resistance to an antibiotic at a final concentration of 20 µg/mL was used, so 20 µL of the antibiotic and 20 mL of agarized LB medium were added [116,129,130].

In the case of the Rosetta strain, 13.6 µL of chloramphenicol (50 mM) was also added at a final concentration of 50 mM. After incubation, 100 µL of the culture was seeded onto the previously prepared plates. These were left to incubate at 37 °C for 24 h to allow the growth of the transformed colonies [130].

DH5α was used for cloning and plasmid amplification because it has a high transformation efficiency, mutations that increase plasmid stability (recA1 and endA1), and supports blue–white screening. It is ideal for obtaining large amounts of high-quality plasmid DNA.

BL21 (DE3) is used for protein expression due to its deficiency in proteases, which reduces the degradation of recombinant proteins and improves yield and stability.

Rosetta (DE3) is strain that also carries a plasmid supplying tRNAs for rare codons in *E. coli*, enabling the efficient expression of eukaryotic or codon-biased genes that otherwise express poorly in standard strains.

### 3.7. Cloning of Plasmid pET-22b with the Inserted Gene

For the cloning of plasmid pET-22b with the inserted gene, a culture was prepared in LB medium. In total, 20 mL of LB medium was added to a 50 mL Falcon tube, which was covered with sterile gauze and Kraft paper to allow adequate oxygenation of the culture. Ampicillin was added to a final concentration of 100 µg/mL, diluting the 100 mg/mL stock at a ratio of 1:1000, which corresponded to the addition of 20 µL of stock solution per 20 mL of culture medium. Subsequently, a colony isolated from the Petri dish previously cultured with the recombinant strain was taken and inoculated into LB medium supplemented with the antibiotic. The culture was incubated at 37 °C with agitation at 220 rpm for 16 h, until the stationary growth phase was reached [57,129,130].

### 3.8. Amplification and Selection of the Transformed Plasmid (Verification of Transformation)

Transformed colonies were selected and subjected to a colony PCR protocol in order to amplify the inserted gene in each colony. PCR reactions were prepared using specific previously designed primers. Subsequently, the amplified products were analyzed by 0.8% agarose gel electrophoresis, running at 100 V for 30 min in 1× TAE buffer. Visualization of DNA bands was performed using ethidium bromide under UV light. The bands corresponding to the amplified fragments were purified using the commercial “Gel Extraction Kit” (Omega BIO-TEK) for subsequent analysis and validation.

### 3.9. DNA Quantification by UV Spectrophotometry

Quantification of genomic and plasmid DNA samples was performed using Nanodrop Spectrophotometer equipment (Thermo Scientific, Waltham, MA, USA). The wavelength used was 260 nm for nucleic acids.

### 3.10. Transformation of BL21 and Rosetta Strains and Amplification of Selected Positive Clones

The plasmid previously transformed and validated by colony PCR was introduced into BL21 and Rosetta strains by electroporation, following the methodology described in this chapter [57,130].

### 3.11. Induction of Protein Expression in the Periplasm by IPTG

After 16 h of growth of the transformed cultures, an aliquot of 100 µL was taken and diluted in 900 µL of LB medium, obtaining a final volume of 1 mL. The optical density (OD) was measured at 600 nm in a spectrophotometer, using LB medium as a blank. Based on OD600, corresponding aliquots were taken and reseeded into 50 mL Falcon tubes. For each 20 mL of culture, 20 µL of ampicillin (100 mg/mL)—and in the case of the Rosetta clones, an additional 13.6 µL of chloramphenicol (50 mM)—was added. The volume was supplemented with LB medium and incubated at 37 °C, with shaking at 220 rpm, until an OD600 of 0.6–0.8 was reached. To induce protein expression, 20 µL of IPTG (1 M) was added per 20 mL of culture, maintaining incubation at 37 °C and 220 rpm for 4 h. After induction, samples were centrifuged at 4 °C, 10,000× *g*, for 10 min. The supernatant was discarded and the pellet was weighed and transferred to an Eppendorf tube. Subsequently, BugBuster Master Mix was added at a ratio of 5 mL per gram of pellet. Samples were shaken for 30 min in a shaker and subsequently centrifuged at 4 °C, 16,000× *g*, for 20 min. The supernatant was collected as the soluble fraction, while the pellet was resuspended again in BugBuster, repeating the shaking and centrifugation process three consecutive times to obtain insoluble fractions 1, 2, and 3 [57,112,130]. The sample was analyzed by electrophoresis in 14% polyacrylamide gel for identification of the chimeric enzyme, Table S2A,B.

The gels were stained with Coomassie Brilliant Blue for 2 h to visualize the proteins, followed by a distinction process using 3 cycles of heating the gel in water for 30 s and then resting for approximately 16 h to visualize the bands.

### 3.12. Quantification of Proteins by UV Spectrophotometry

Total protein quantification of the samples was carried out using Nanodrop Spectrophotometer equipment (Thermo Scientific). The wavelength used was 280 nm.

### 3.13. Detection of Asparaginase Activity by Qualitative Hydroxylamine Assay

For the performance of the qualitative hydroxylamine assay, a mixture was prepared starting from 700 μL of 50 mM Tris-HCl buffer, pH 8.8. To this solution, 100 μL of 1 M hydroxylamine, pH 7, followed by 100 μL of 100 mM asparagine, was added sequentially. Subsequently, 100 μL of the sample under evaluation was incorporated and the mixture was incubated at 37 °C for 30 min. After this time, 250 μL of ferric chloride (100 mg/mL) was added for the detection of enzyme activity. The appearance of a characteristic coloration indicated the presence of asparaginase (ASNase) activity [57].

### 3.14. Measurement of Activity by Nessler’s Assay

The SIGMA QUALITY CONTROL TEST PROCEDURE ENZYMATIC ASSAY OF ASPARAGINASE (EC 3.5.1.1) assay adapted to microplates was followed.

### 3.15. Optimization of the Process of Induction of Chimeric Protein Expression by IPTG

In order to optimize the induction of chimeric protein expression by IPTG, an analysis based on an experimental design was carried out using the statistical software Design-Expert 13. For this purpose, a response-surface model was used, evaluating the effect of three key variables: induction temperature, IPTG concentration, and incubation time Table S3A. The experimental conditions evaluated are presented in Table 1.

### 3.16. Scale-Up of the Culture to 1L and Induction of Protein Expression in the Periplasm by IPTG

Following the protocol previously described in the methodology, the culture was pre-inoculated, allowing its growth for 16 h. Subsequently, an aliquot of 100 µL was taken and diluted in 900 µL of LB medium, obtaining a final volume of 1 mL. The optical density (OD) was measured at 600 nm in a spectrophotometer, using LB medium as a blank control. The volume of the culture was adjusted according to its OD and seeded in a 1 L Erlenmeyer flask, to which 100 mL of the culture, 1000 µL of ampicillin and, in the case of the Rosetta clones, 680 µL of chloramphenicol were added. The total volume was adjusted to 1 L with LB medium and incubated at 37 °C with shaking at 220 rpm until an OD of 0.6 to 0.8 was reached. Recombinant protein expression was induced by the addition of 500 µL of IPTG, reaching a final concentration of 0.5 mM [130]. Incubation was maintained at 37 °C with shaking at 220 rpm for 4 h. Subsequently, the samples were centrifuged at 4 °C at 10,000× *g* for 10 min, discarding the supernatant. The pellet obtained was weighed and transferred to an Eppendorf tube for further processing [57].

### 3.17. Protein Extraction

#### Sonication

Lysis buffer (according to the composition detailed in Table S4) was added to the cell pellet at a ratio of 30 mL per pellet from 1 L of culture. The pellet was completely resuspended and then subjected to sonication for 10 min, using a stem sonicator, using a 5 s pulse cycle of sonication with 10 s rest intervals, at 50% power [57].

After sonication, the samples were centrifuged at 4 °C at 10,000× *g* for 20 min. The supernatant was recovered and stored as the soluble protein fraction.

### 3.18. Purification of the Engineered Chimeric Protein

#### AKTA Star Affinity Chromatography

The purification process of the designed chimeric protein was carried out by nickel-affinity chromatography using HisTrap™ HP columns (GE Healthcare Life Sciences, Little Chalfont, Buckinghamshire, UK) on an AKTA Star chromatographic system. Initially, the cell-free extract obtained after sonication was clarified by filtration through a 0.22 µm filter for the removal of cell debris and other contaminants. The affinity columns were previously equilibrated with buffer A, composed of 50 mM sodium phosphate, 300 mM NaCl, and 100 mM imidazole, adjusted to a pH of 7.4, in order to prepare the matrix for the selective capture of histidine-tagged proteins (His-tag). Once the sample was applied to the column, it was washed with buffer A to remove proteins nonspecifically bound to the resin.

Elution of the protein of interest was carried out by applying an increasing gradient of imidazole, with concentrations in the range of 100 to 500 mM in a buffer solution composed of 50 mM sodium phosphate and 300 mM NaCl, with a pH of 7.4 [57]. This procedure allowed the specific dissociation of the recombinant protein bound to the nickel resin, facilitating its recovery in differentiated fractions for subsequent analysis and characterization. To evaluate the efficiency of the chromatography, polyacrylamide gel electrophoresis was performed, and a NanoPore [112] was additionally used to determine the amount of total proteins. The methodologies previously described were followed.

### 3.19. 12% Polyacrylamide Gel Electrophoresis for Identification of the Chimeric Enzyme

Separator and concentrator gels were prepared following the standard formulation described below in Table S5. A 10 µL aliquot of sample were taken and mixed with 10 µL of loading buffer. Electrophoresis was carried out at 100 V for 2 h. Subsequently, the gels were stained with Coomassie Brilliant Blue for 2 h for protein visualization, followed by a deinking process using an acetic acid and methanol solution.

### 3.20. Protein Identification by Western Blot

Protein electrotransfer from the polyacrylamide gel to a nitrocellulose membrane was carried out, ensuring the correct alignment and labeling of the lane corresponding to the molecular weight marker. The transfer was performed by the wet method, assembling the transfer chamber with blot buffer and applying a voltage of 80–100 V for 1 h in an ice bath to minimize overheating. Subsequently, the membrane was blocked with 5% nonfat milk in 1× PBS (pH 7.4) for 1 h with shaking at room temperature, followed by incubation with primary antibody in 1% nonfat milk in 1× PBS (pH 7.4) at 4 °C overnight. Five consecutive washes with Tween-20 in 1× PBS (pH 7.4) were performed before incubation with the secondary antibody under the same conditions for 2 h at room temperature, followed by another five washes with the same solution. Detection was carried out by chemiluminescence using the SuperSignal™ West Pico PLUS substrate (Thermo Scientific™, Waltham, MA, USA), and images were obtained with the G:Box Chemi XRQ system (Syngene, Cambridge, UK), ensuring proper signal registration. As an alternative method of protein identification in this experiment, a goat anti-mouse HRP (horseradish peroxidase)-conjugated anti-His antibody was used.

### 3.21. Statistical Analysis

#### Full Factorial Design

To study the effect of three factors—induction time, culture temperature, and IPTG concentration—on the total enzymatic activity of L-asparaginase (U-L^−1^), a 2^3^ full factorial design was used. This design allows the evaluation of the main effects of each variable, as well as their first-order interactions, using two levels for each factor: a low level (−1) and a high level (+1). The design consisted of 8 basic experimental combinations, to which central points (n = 4) were added in order to verify the linearity of the model and to detect possible curvature effects. Experiments were performed randomly to minimize experimental bias. Enzyme activity was determined for each experimental condition and the results obtained were processed using Design Expert software version 13 (Stat-Ease Inc., Minneapolis, MN, USA), Table S3B. To determine statistically significant effects (*p*-value ≤ 0.05), an analysis of variance (ANOVA) was performed. This analysis also made it possible to evaluate the adequacy of the model by means of the lack of fit test.

The standard curve for activity determination by the Nessler method is in Supplementary Figure S5.

## 4. Conclusions

The successful design, expression, and purification of a humanized chimeric L-asparaginase enzyme were achieved by combining fragments from *Escherichia coli* and *Homo sapiens* ASNase. This approach retained enzymatic activity while incorporating less immunogenic regions from the human enzyme. The *E. coli* Rosetta strain proved more effective than BL21 in achieving soluble and functional expression of the chimeric enzyme, with enzymatic activity reaching up to 2.4 U/mL after process optimization. Activity assays using the hydroxylamine and Nessler methods confirmed that the chimeric protein retains its enzymatic functionality, as evidenced by both qualitative and quantitative results, suggesting correct folding in the periplasmic environment. Furthermore, response-surface methodology was employed to optimize induction conditions, identifying 37 °C, 0.01 mM IPTG, and 2 to 4 h of induction time as the optimal parameters for maximizing activity. The enzyme was successfully purified via nickel-affinity chromatography (His-tag), achieving high purity in a single step, as shown by the chromatogram and the absence of secondary peaks. However, future studies will focus on confirming the periplasmic localization of the chimeric enzyme, which is expected to enhance proper folding and functional activity. Establishing this will be essential to validate the advantages of periplasmic expression for improving enzyme stability and therapeutic potential. Such investigations will strengthen the foundation for developing this enzyme as a safer and more effective biopharmaceutical.

Looking ahead, several perspectives emerge from this work. First, in vitro and in vivo immunological testing is recommended to confirm reduced immunogenicity and to verify the immune tolerance of the humanized chimeric variant using human lymphocytes and animal models. Second, detailed kinetic characterization, including the determination of KM, Vmax, and substrate specificity, is needed to benchmark the enzyme’s catalytic efficiency against commercial ASNase variants. Third, evaluating the enzyme’s thermal and storage stability, as well as scaling up production to a pilot level, will be essential steps for assessing its potential as a biopharmaceutical. Fourth, genetic construct improvements—such as further optimization of the pelB signal peptide—may enhance periplasmic secretion and correct protein folding. Finally, future work should involve comparative clinical evaluations between this chimeric enzyme and clinically approved ASNase formulations, like PEG-asparaginase, focusing on therapeutic efficacy, toxicity, and immunogenic profiles.

## Figures and Tables

**Table 1:** Experimental design of the optimization process of chimeric asparaginase expression. The variables that were evaluated and the values corresponding to each one are shown, as well as the randomized design. The table shows STD (standard order), Run (run order), time, IPTG (isopropyl β-D-1-thiogalactopyranoside), and temperature.

STD	Run	Time	IPTG [mM]	Temperature °C
13	3	6	0.505	20
1	4	4	0.2575	24.25
2	6	8	0.2575	24.25
3	16	4	0.7525	24.25
4	2	8	0.7525	24.25
9	13	2	0.505	28.5
10	19	10	0.505	28.5
11	5	6	0.01	28.5
12	17	6	1	28.5
15	15	6	0.505	28.5
16	20	6	0.505	28.5
17	10	6	0.505	28.5
18	12	6	0.505	28.5
19	1	6	0.505	28.5
20	14	6	0.505	28.5
5	8	4	0.2575	32.75
6	18	8	0.2575	32.75
7	7	4	0.7525	32.75
8	11	8	0.7525	32.75
14	9	6	0.505	37

## Data Availability

Data is contained within the article and Supplementary Materials.

## Supplementary Materials

The following supporting information can be downloaded at https://www.mdpi.com/article/10.3390/ijms26146919/s1.
