# Supplementary material for: Toward Safer Biotherapeutics: Expression and Characterization of a Humanized Chimeric L-Asparaginase in E. coli
Source: Int J Mol Sci. 2025 Jul 18;26(14):6919. doi: 10.3390/ijms26146919 (PMC12295240; doi:10.3390/ijms26146919)
Supplement: Supplementary file 1 [file ijms-26-06919-s001.zip › ijms-3712353-supplementary.pdf]

Data for the cloning of the Chimeric Asparaginase

Optimized sequence ASN_H_Q

CTG CCT AAC ATA ACC ATT TTG GCA ACG GGC GGT ACT ATC GCG GGC
GGA GGG GAT TCA GCG ACT AAG AGC AAC TAT ACC GTG GGT AAG
GTG GGG GTA GAC GCT GTC GAG GGG GCA GTC GTC GCA CTT GAA GAT
GAT CCG AAC GTG AAA GGA GAG CAG GTG GTA AAC ATT GGC TCC
CAA GAT ATG AAC GAC AAC GTG TGG TTG ACT TTG GCG AAG AAG ATC
AAC ACA GAC TGT GAC AAG ACG GAT GGG TTT GTA ATA ACA CAT GGG
ACC GAT ACA ATG GAA GAA ACG GCG TAT TTC TTG GAC TTA ACG GTG
AAA TGT GAC AAG CCC GTC GTA ATG GTC GGT GCT ATG AGA CCT AGT
ACG AGC ATG AGC GCG GCG AAT CCT ATC AAA TTG GCT AGA TTA GTA
ATG GAA AAG ACT CCC AAG GCT AGT GCG AAT CGT GGC GTC CTG GTC
GTA ATG AAT GAC ACG GTA TTG GAT GGT CGG GAT GTA ACA AAG ACG
AAC ACC ACT GAC GTT GCC ACG TTT AAG TCC GTA AAC TAC GGG CCA
TTA GGA TAT ATA CAT AAC GGG AAA ATC GAT TAT CAG CGC ACG CCA
GCA CGC AAA CAC ACTTCG GAC ACG CCC TTT GAC GTC AGT AAG CTG
AAT GAA TTA CCA AAA GTC GGA ATC GTG TAC AAC TAC GCA AAC GCA

AGC GAC CTT CCC GCG AAA GCG TTG GTT GAC GCG GGG TAT GAC GGT
ATC GTT TCA GCC GGA GTA GGG AAC GGC AAT TTA TAC AAG TCC GTA
TTT GAC ACG CTT GCA ACA GCA GCG AAA ACT GGG ACA GCA GTA GTC
CGG TCT AGC CGT GTC CCA ACA GGA GCA ACA ACC CAA GAT GCG
GAA GTC GAC GAT GCC AAG TAC GGG TTC GTG GCT TCT GGA ACC TTG
AAC CCA CAA AAG GCT CGC GTT TTG CTT C AG CTG GCG CTG ACT CAG
ACC AAG GAT CCG CAG CAA ATA CAG CAA ATC TTC AAC C AA TAT

Secuencia de vírus del tabaco Etch (TEV)

E N L Y F Q* G

gaa aac ctg tat ttt cag ggc

Histidine sequences

H H
H H
H H

gtg gtg gtg gtg Gtg gtg

Primers His 6x + TEV

N-terminal histidine tag

ASNasa_H_Q_OPT_NcoI_ FW 5´ggc GGT CTC c<u>CA TGg g</u>c**c acc acc acc acc acc**
acg aaa acc tgt att ttc agg gcC TG CCT AAC ATA ACC ATT TTG 3´

GC CONTENT	48.3 %
GC CONTENT	54.5 %
MELT TEMP	73.5 ºC
MELT TEMP	72 ºC

ASNasa_H_Q_OPT_ XhoI_ RV 5´ **ggc GGT CTC cCT CGA Gtc Aata ttg gtt gaa gat ttg ctg tat ttg ctg cgg atc ctt GGT CTG AGT cag cg 3´**

Expressed protein
HHHHHHENLYFQ*GLPNITILATGGTIAGGGDSATKSNYTVGKVGVDAVEGAV
VALEDDPNV
KGEQVVNIGSQDMNDNVWLTLAKKINTDCDKTDGFVITHGTDTMEETAYFLDL
TVKCDKPVV
MVGAMRPSTSMSAANPIKLARLVMEKTPKASANRGVLVVMNDTVLDGRDVTK
TNTTDVATF
KSVNYGPLGYIHNGKIDYQRTPARKHTSDTPFDVSKLNELPKVGIVYNYANASD
LPAKALVDA
GYDGIVSAGVGNGNLYKSVFDTLATAAKTGTAVVRSSRVPTGATTQDAEVDDA
KYGFVASGT
LNPQKARVLLQLALTQTKDPQQIQQIFNQY
*: Cleavage by the tobacco virus protease.

A .

LPNITILATGGTIAGGGDSATKSNYTVGKVGVENLVNAVPQLKDIANVK
GEQVVNIGSQDMNDNVWLTLAKKINTDCDKTDGFVITHGTDTMEET
AYFLDLTVKCDKPVVMVGAMRPSTSMSADGPFNLYNAVVTAADKASA
NRGVLVVMNDTVLDGRDVTKTNTTDVATFKSVNYGPLGYIHNGKIDY
QRTPARKHTSDTPFDVSKLNELPKVGIVYNYANASDLPAKALVDAGYD
GIVSAGVGNGNLYKSVFDTLATAAKTGTAVVRSSRVPTGATTQDAEVD
DAKYGFV ASGTLNPQKARVLLQLALTQTKDPQQIQQIFNQY

B.

HMNPIVVVHGGGAGPISKDRKERVHQGMVRAATVGYGILREGGSAV
DAVEGAVVALEDDPEFNAGCGSVLNTNGEVEMDASIMDGKDLSAGA
VSAVQCIANPIKLARLVMEKTPHCFLTDQGAAQFAAAMGVPEIPGEKL
VTERNKKRLEKEKHEKGAQKTDCQKNLGTVGAVALDCKGNVAYATSTG
GIVNKMVGRVGDSPCLGAGGYADNDIGAVSTTGHGESILKVNLARLTL
FHIEQGKTVEEAADLSLGYMKSRVKGLGGLIVVSKTGDWVAKWTSTS
MPWAAAK DGKLHFGIDPDDTTITDLP

C.

LPNITILATGGTIAGGGDSATKSNYTVGKVGVDAVEGAVVALEDDPNV
KGEQVVNIGSQDMNDNVWLTLAKKINTDCDKTDGFVITHGTDTMEE
TAYFLDLTVKCDKPVVMVGAMRPSTSMSAANPIKLARLVMEKTPKAS
ANRGVLVVMNDTVLDGRDVTKTNTTDVATFKSVNYGPLGYIHNGKID
YQRTPARKHTSDTPFDVSKLNELPKVGIVYNYANASDLPAKALVDAGYD
GIVSAGVGNGNLYKSVFDTLATAAKTGTAVVRSSRVPTGATTQDAEVD
DAKYGFV ASGTLNPQKARVLLQLALTQTKDPQQIQQIFNQY

Figure S1.A) Amino acid sequence of native E. coli L-asparaginase (3ECA) and human L-asparaginase (4O0H);B) Human L-asparaginase sequence (4O0H);C) Chimeric ASNase_H_Q sequence.Human protein fragments that were inserted into the E. coli 3ECA sequence are highlighted in blue. Regions selected for substitution are shown in green and red

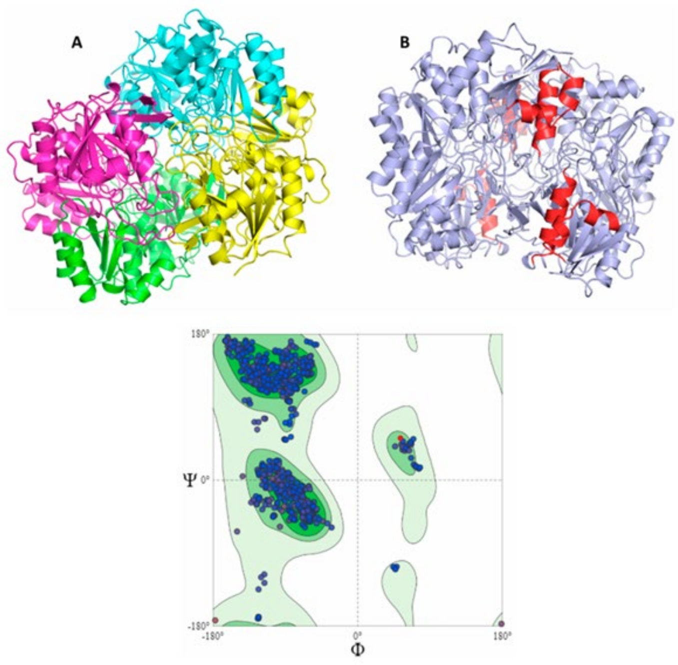

(C)

Figure S2. Humanized chimeric E. coli L-asparaginase with fragments of human L-asparaginase 4O0H and evaluation of the accuracy of the predicted protein structure. (A) The humanized 3ECA-derived Figure 3. Humanized chimeric E. coli L-asparaginase with fragments of human L-asparaginase 4O0H and evaluation of the accuracy of the predicted protein structure. (A) The humanized 3ECA derived chimeric enzyme is shown in tetrameric conformation with four independently folded sub units. Subunit A is shown in green, subunit B in cyan, subunit C in magenta, and subunit D in yel low. (B) The humanized 3ECA-derived chimeric enzyme is shown; the fragments of human L-as paraginase 4O0H that were substituted are shown in red. (C) Ramachandran diagram for the chi meric L-asparaginase protein. Observed values of the Φ and Ψ angles (blue point) for all humanized 3ECA-derived amino acids in the chimeric protein. In dark green are presented the allowed and favorable zones. In light green, the less favorable zones are presented and in lighter green and white, chimeric enzyme is shown in tetrameric conformation with four independently folded subunits. (B)The humanized 3ECA-derived chimeric enzyme is shown; the fragments of human L-asparaginase 4O0H that were substituted are shown in red. (C) Ramachandran diagram for the chimeric L-asparaginase protein. Observed values of the and angles (blue point) for all humanized 3ECA-derived amino acids in the chimeric protein. In dark green are presented the allowed and favorable zones. In light green, the less favorable zones are presented and in lighter green and white, the most unstable and prohibited areas, respectively, where the combinations of the and angles are not allowed by steric hindrance. In the first quadrant, the left-helix combinations are represented, in the second quadrant are the-sheet combinations, and in the third quadrant are the right-helices and loops. The chimeric protein was characterized by a stable conformation with most of the amino acids in the allowed zone. It was characterized by a predominance of right-helices and-sheets. **(73)**

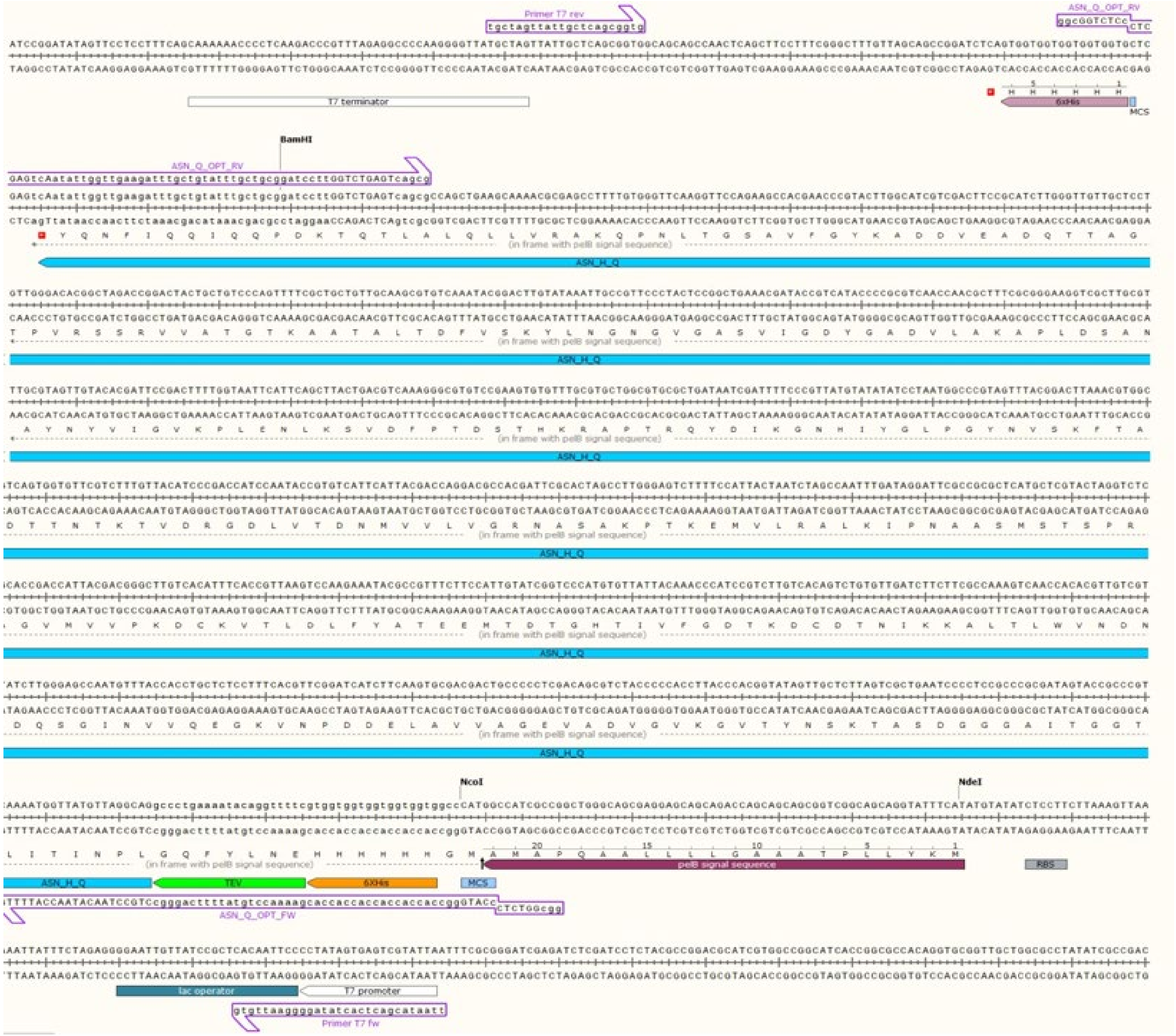

Figure S3. Map of the linear expression vector

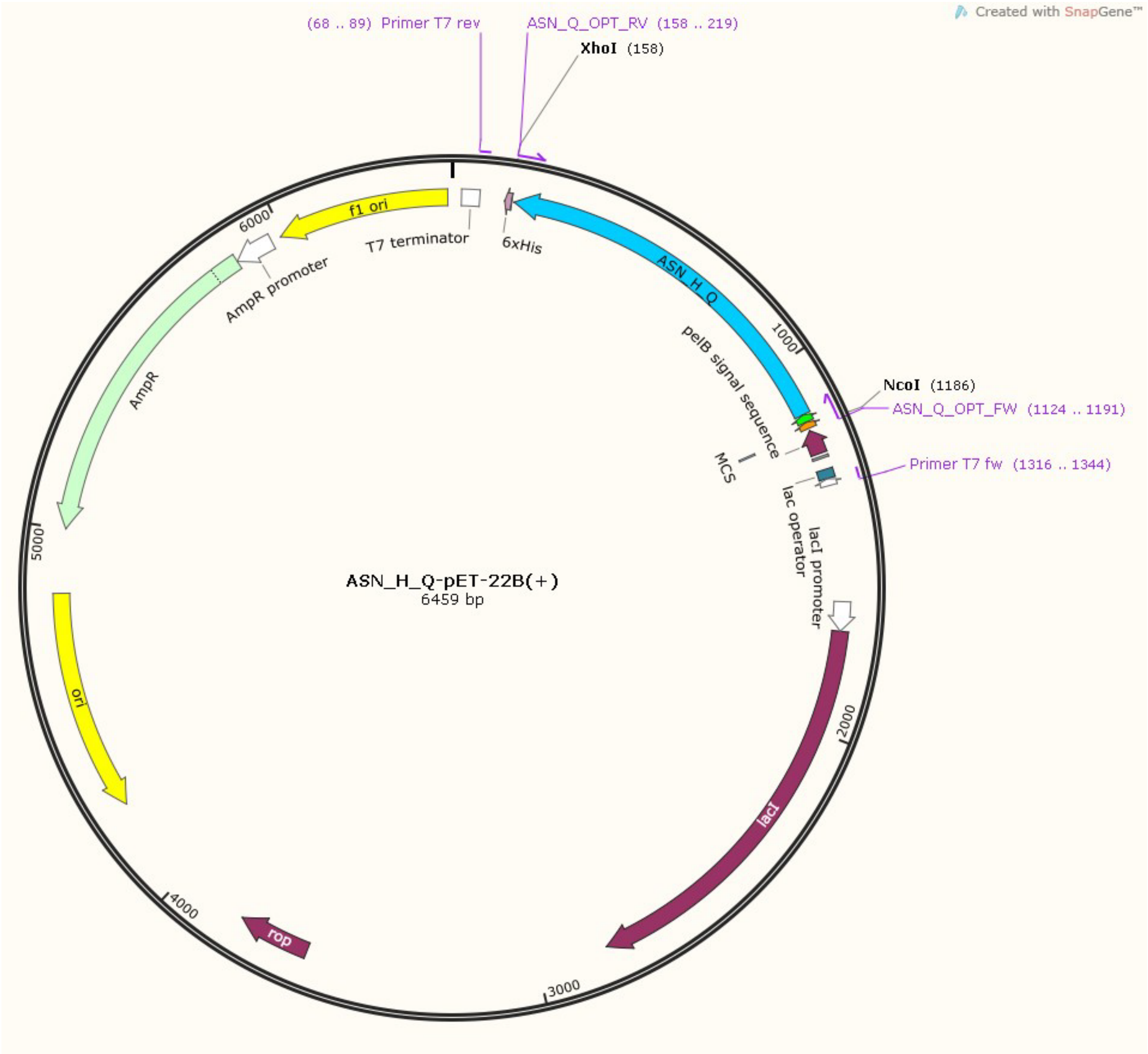

Figure S4. Map of the circular expression vector

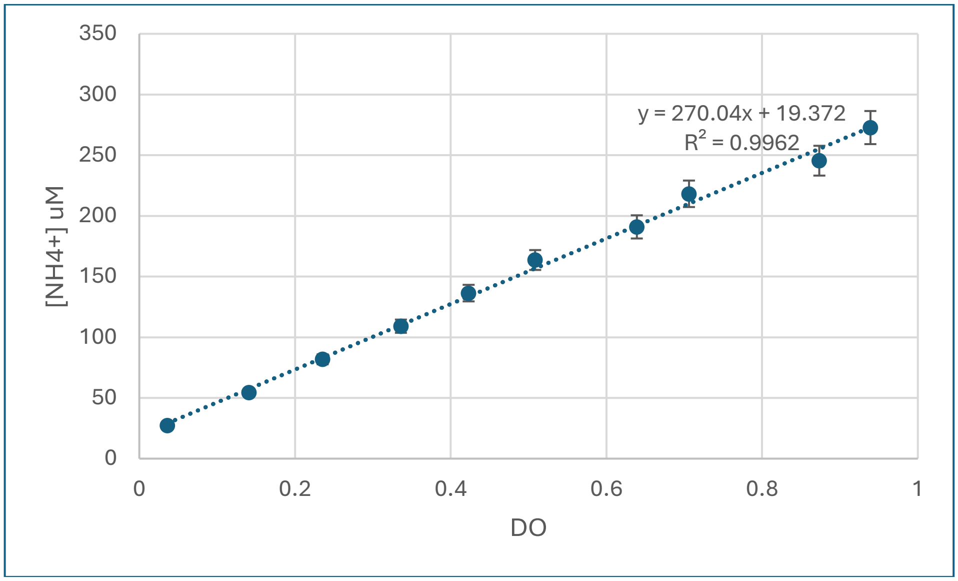

Figure S5. Standard curve for activity determination by the Nessler method

Table S1. Content of master mix used to perform conventional PCR.

Compound	1X (Volumes in µL)
10X Buffer	10 µL
dNTPs	5 µL
ASNase Gene (10 ng/µL)	5 µL
Primers (2.5 µM)	10 µL
DMSO (optional)	1.5 µL
Polymerase	0.5–1 µL
Water	18 µL

Table S2A Electrophoresis in 14% polyacrylamide gel for identification of the chimeric enzyme (separator and concentrator gels were prepared as described below):Samples were prepared by soluble and insoluble fraction 1, 2 and 3 as described in Table 2:

SDS-PAGE 14% (1 mm)	
**Separation Gel**	**Packing Gel**
- 2.8 mL 1M Tris-HCl buffer pH 8.8<br>- 3 mL 30% polyacrylamide<br>- 10 µL TEMED<br>- 50 µL 10% PSA	- 400 µL 1M Tris-HCl buffer pH 6.8<br>- 500 µL 30% polyacrylamide<br>- 2.5 mL H_2O<br>- 5 µL TEMED<br>- 25 µL PSA 10%

Table S2B. Sample preparation for 14% SDS- PAGE electrophoresis.

Component (µL)	Soluble Sample	Insoluble 1	Insoluble 2	Insoluble 3
Solution	10	10	10	10
MilliQ Water	10	10	10	10
SDS	5	5	5	5
DTT	2	2	2	2

Table S3A. Full factorial design 2^3

STD	RUN	Time	IPTG (mM)	Temp (°C)	Activity (U/mL)
13	3	6	0,505	20	0,681
1	4	4	0,2575	24,25	0,833
2	6	8	0,2575	24,25	0,471
3	16	4	0,7525	24,25	0,421
4	2	8	0,7525	24,25	1,362
9	13	2	0,505	28,5	0,649
10	19	10	0,505	28,5	0,691

11	5	6	0,01	28,5	0,805
12	17	6	1	28,5	0,869
15	15	6	0,505	28,5	0,901
16	20	6	0,505	28,5	0,926
17	10	6	0,505	28,5	1,002
18	12	6	0,505	28,5	0,637
19	1	6	0,505	28,5	0,510
20	14	6	0,505	28,5	0,817
5	8	4	0,2575	32,75	0,621
6	18	8	0,2575	32,75	0,899
7	7	4	0,7525	32,75	0,645
8	11	8	0,7525	32,75	0,770
14	9	6	0,505	37	1,954

Table S3B. ANOVA analysis of the effect of temperature, time, and IPTG concentration on the total activity of chimeric L-ASNase obtained from the full factorial design 2^3

Source of Variation	Degrees of Freedom (DF)	Sum of Squares (SS)	Mean Square (MS)	F-Statistic	p-Value
Model	14	0.9699	0.0693	2.45	0.0545
Time (A)	1	0.1002	0.1002	3.54	0.1568
IPTG Concentration (B)	1	0.1026	0.1026	3.63	0.6788
Temperature (C)	1	0.0044	0.0044	0.16	0.4926
AB	1	0.0007	0.0007	0.02	0.8755
AC	1	0.0016	0.0016	0.05	0.8198
BC	1	0.0002	0.0002	0.01	0.9091
ABC	1	0.0785	0.0785	2.70	0.0299
A^2	1	0.1563	0.1563	5.37	0.0653
B^2	1	0.0136	0.0136	0.47	0.5422
C^2	1	0.1211	0.1211	4.16	0.1031
Error	6	0.0733	0.0122		
Total	20	1.0432			

Table S4. Chemical composition of the lysis buffer.

Compound	Concentration
Tris HCl	100 mM
Sodium Phosphate	100 mM
NaCl	100 mM
DNase	5 µg/mL
RNase	5 µg/mL
Lysozyme	1 µg/mL
PMSF	0.5 mM
Glycerol	5 %

Table S5. 12% SDS-PAGE electrophoresis preparation.

Buffers	12% Separating Gel	4% Stacking Gel
Upper	-	0.625 mL
Lower	1.25 mL	-
Acrylamide/Bisacrylamide 30%	2.04 mL	0.325 mL
Distilled H2O	1.625 mL	1.525 mL
SDS 10%	50 µL	25 µL
PSA 10%	75 µL	30 µL
TEMED	10 µL	10 µL
